# Separation of extracellular nanovesicles and apoptotic bodies from cancer cell culture broth using tunable microfluidic systems

**DOI:** 10.1038/s41598-017-08826-w

**Published:** 2017-08-30

**Authors:** Soojeong Shin, Daeyoung Han, Min Chul Park, Ji Young Mun, Jonghoon Choi, Honggu Chun, Sunghoon Kim, Jong Wook Hong

**Affiliations:** 1 Department of Bionano Engineering, Hanyang University, Ansan, 15588 Korea; 2 Medicinal Bioconvergence Research Center, Seoul National University, Seoul, 08826 Korea; 3 Department of Molecular Medicine and Biopharmaceutical Sciences, Graduate School of Convergence Technology, College of Pharmacy, Seoul National University, Seoul, 08826 Korea; 4 Advanced Institutes of Convergence Technology, Seoul National University, Suwon, 16229 Korea; 5 Department of Biomedical Laboratory Science, College of Health Sciences, Eulji University, Seongnam, 13135 Korea; 6 BK21 Plus Program, Department of Senior Healthcare, Graduate School, Eulji University, Daejeon, 34824 Korea; 7 School of Integrative Engineering, Chung-Ang University, Seoul, 06974 Korea; 8 Department of Biomedical Engineering, Korea University, Seoul, 02841 Korea; 9 Department of Bionano Technology, Graduate School, Hanyang University, Seoul, 04763 Korea

## Abstract

Extracellular vesicles (EVs) are the cell-secreted nano- and micro-sized particles consisted of lipid bilayer containing nucleic acids and proteins for diagnosis and therapeutic applications. The inherent complexity of EVs is a source of heterogeneity in various potential applications of the biological nanovesicles including analysis. To diminish heterogeneity, EV should be isolated and separated according to their sizes and cargos. However, current technologies do not meet the requirements. We showed noninvasive and precise separation of EVs based on their sizes without any recognizable damages. We separated atto-liter volumes of biological nanoparticles through operation of the present system showing relatively large volume of sample treatment to milliliters within an hour. We observed distinct size and morphological differences of 30 to 100 nm of exosomes and apoptotic bodies through TEM analysis. Indeed, we confirmed the biological moiety variations through immunoblotting with noninvasively separated EVs opening new windows in study and application of the biological nanoparticles.

## Introduction

The complexity of a biological system can be resolved by using its physicochemical properties to isolate and separate its specific constituent bio-organisms and -molecules, such as cells, bacteria, proteins, and DNA. Recently, there is an emerging need to isolate and separate extracellular vesicles (EVs). EVs are lipid bilayer vesicles secreted by cells, and generally, the size ranges between hundreds of nanometers. Since EVs contain specific biomolecules such as protein, mRNA, and microRNA, their potential application in diagnostics and therapeutics^1–3^ has garnered considerable attention.

Besides the size and density, EVs’ heterogeneity also derives from the diverse cargo inserted in EVs, making it arduous for researchers to determine their exact functions^4^. Based on accumulated evidence, EVs are classified into exosomes, microvesicles, and apoptotic bodies^4^. Of these, exosomes, a well-characterized EV type, are of particular interest to the medical and pharmaceutical fields^5^. Exosomes are membranous vesicles of endosomal origin with 50–200 nm hydrodynamic diameter and have biologically significant molecules^6^.

However, a validated protocol for the isolation of these small vesicles with diameters of approximately 100 nm has not been suggested yet. The most commonly used method is ultracentrifugation, which is time and energy consuming, and has the risk of introducing protein and other types of EV contamination because of a highly pressurized environment^7, 8^. Alternative methods such as size exclusion chromatography^9, 10^, filtration^11^, precipitation^12^, and flow-cytometric analysis^13^ also suffer from their own limitations.

Miniaturized fluidic channel offers a potential means for experimental biological research^14–16^, including exosome separation^17–19^. Microfluidics builds upon various physicochemical parameters, such as chemical binding for antibody application^20, 21^, sieving^22^, acoustic wave^23^, and field flow fractionation^24^ onto micro- and nano-scale devices, which show high accuracy, precise control, lower energy consumption, and minimal sample size. Reported EV separating microdevices, however, have a narrow size range of sortable EVs, requiring additional pretreatment steps, which may cause sample dysfunction. Moreover, affinity-based EV separation catches only specific targets, thus missing unknown biological nanoparticles of potential value.

**Figure 1:**
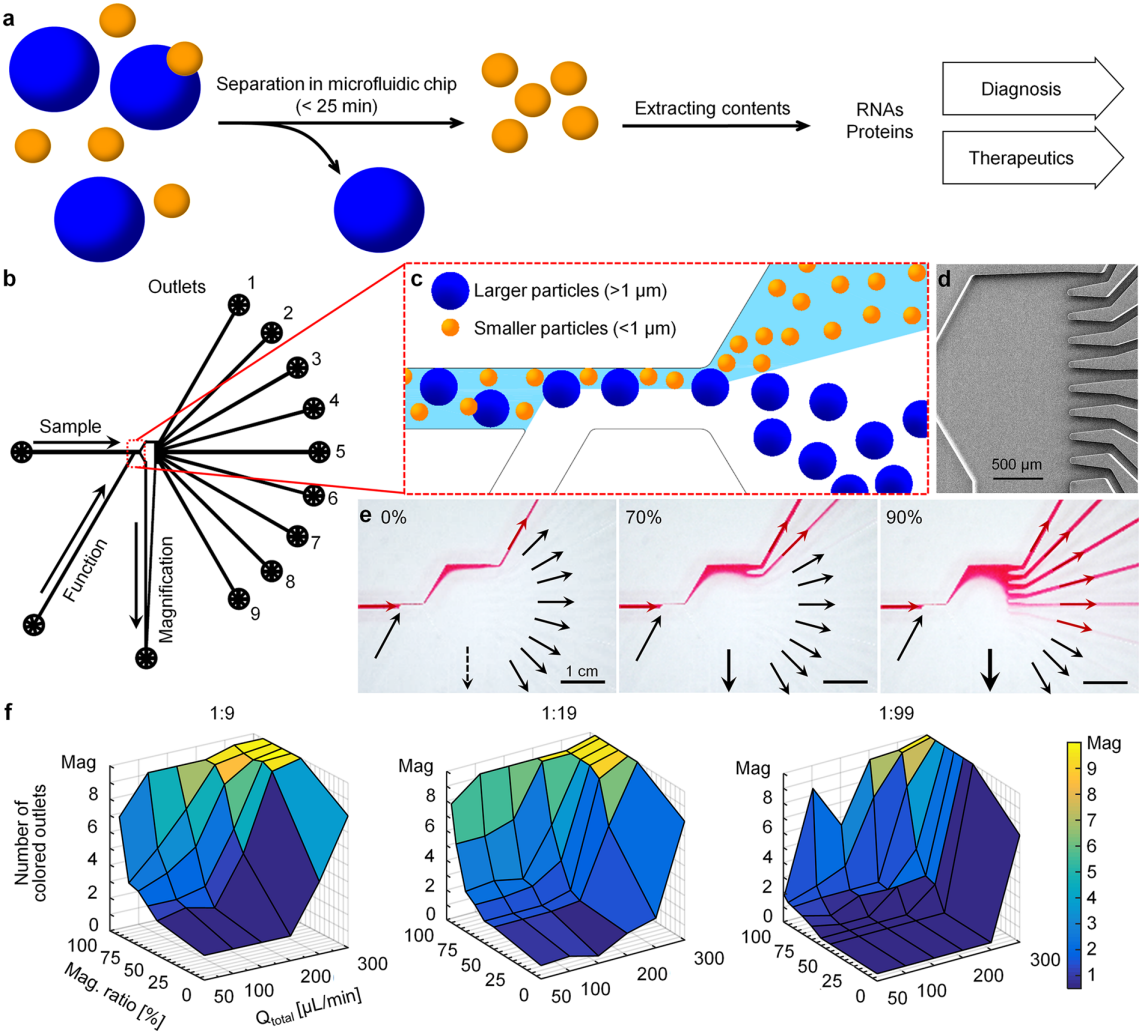
Microfluidic chip design and operational conditions. (**a**) Our goal is to separate unpurified biological nano-vesicles and/or micro-particles under mild external force. Since separated vesicles/particles are merely damaged during the process, the present methodologies and systems can be applied not only to diagnosis but also to therapeutics. (**b**) Microchannel design consisting of two inlets (Sample and Function channels), nine outlets (numbered from #1 to #9), and a magnification channel that withdraw the flow. (**c**) (Top view) Schematic diagram of size separation of nano-vesicles and micro-particles at the core region of microfluidic device (not to scale). Nano-vesicles and micro-particles are aligned through the upper wall. Then, larger vesicles/particles move toward outlets near the magnification channel while smaller ones travel to upper outlets when channel width is broadened. (**d**) Scanning electron microscopy (SEM) of the core part of the chip. (**e**) Pictures of sophisticate control of Sample flow from outlet channel 1 to 9 as a function of withdrawal speed: Under Sample:Function ratio is 1:19, 0, 70, and 90% of total flow is withdrawn to the magnification channel. Red dye represents Sample flow. Increased flow in withdrawing magnification channel results in spreading of Sample flow through outlet channels, #1 to #9. (**f**) Three-dimensional landscaped graphs depict relations among *Q*_*total*_, magnification ratio (MR), and the number of outlets filled with Sample fluid under the different Sample:Function ratios, 1:9, 1:19, and 1:99. The spread area of Sample flow in the core part of the chip is increased as *Q*_*total*_ and MR increase.

Here, we report noninvasive size-based EV separation on a chip and analyze separation patterns of micro- and nano-vesicles/particles. We used a microfluidic device to perform EV separation based on heterogeneous sizes with diameters between 0.1 and 5 μm. The suggested separation system requires less than an hour, with minimized external force, and can be an alternative method for EV evaluation (Fig. 1a). We applied various sizes of polystyrene (PS) particles as well as cell-cultured media containing different vesicles. Newly proceeded vesicle/particle separation methods as well as significant results on diagnosis were discussed.

## Results

### An effect of magnification ratio in microchannel for EV separation

The designed microchannel is depicted in Fig. 1b. The microdevice is composed of two inlets (Sample and Function channel), nine outlets, and a magnification channel that controls the overall flow pattern. Ideally, the Sample and Function flows come in contact inside the channel, then the pinched channel expands by 21 fold, allowing for spaces between vesicles/particles to increase perpendicular to the direction of the flow (Fig. 1c). The magnification channel draws part of the Function flow and any remaining Function and Sample flows are directed towards their respective outlets.

The vesicle/particle allocation could be altered as changing the ratio of flow withdrawn to overall inserted flow through the magnification channel, named as the magnification ratio (MR [%] = *Q*_*M*_/*Q*_*total*_ × 100), where *Q*_*M*_ and *Q*_*total*_ are magnification flow rate and total flow rate, respectively. In the absence of magnification, meaning 0% MR, all Sample flow (red dye in Fig. 1e) ran to the first outlets and no significant change of colored outlet was observed until 50% MR was attained (Supplementary Fig. S2). When the MR increased to 70–90% under the 1:19 of Sample:Function ratio (Fig. 1e), the number of outlet channels containing Sample flow increased significantly. Additionally, effects of Sample:Function ratio and *Q*_*total*_ as well as MR were evaluated comprehensively. Yellowish areas at Fig. 1f represent partial loss of Sample flow withdrawn to the magnification channel rather than outlets #1 to #9, indicating over-magnification and commonly observed at *Q*_*total*_ ≥ 250 μL/min and high MR (≥80% at 1:9, ≥90% at 1:19, ≥95 at 1:99). Especially, the dye is spread more than the theoretically expected values (Table 1) when *Q*_*total*_ > 200 μL/min. On the other hand, dark blue areas at Fig. 1f indicate insufficient Sample flow broadening, which induce non-separated particle outflow. This kind of phenomena appeared when MR was lower than 60% at 1:9 and 80% at 1:19 when *Q*_*total*_ was fixed under 200 μL/min. With the ratio of 1:99 of Sample:Function, MR is more than 80% for every *Q*_*total*_ condition. Based on these results, *Q*_*total*_ between 50–200 μL/min, MR between 60–90%, and 1:9 and 1:99 of Sample:Function ratios were set for the rest of the experiments.

**Table 1:** Number of colored outlets according to the magnification ratio (MR), *Q*_*total*_, and Sample:Function ratios.

Magnification ratio [%]	Number of colored outlets																				
	theoretical			50 μL/min			100 μL/min			150 μL/min			200 μL/min			250 μL/min			300 μL/min		
	A*	B*	C*	A	B	C	A	B	C	A	B	C	A	B	C	A	B	C	A	B	C
0	0.9	0.5	0.1	1.5	0.9	0.5	1.5	1.0	0.5	1.5	0.5	0.5	1.5	1.5	0.5	4.4	2.0	0.5	8.0	8.0	7.2
50	1.8	0.9	0.2	2.1	1.5	0.5	2.5	1.5	0.5	2.1	1.5	0.5	5.0	1.7	0.5	9.6	6.2	0.5	10	10	10
60	2.3	1.1	0.2	2.7	1.5	0.5	2.7	1.5	0.5	3.1	1.5	0.5	6.2	2.0	0.5	9.9	9.0	2.0	10	10	10
70	3.0	1.5	0.3	3.3	2.2	0.9	4.0	2.2	0.5	5.2	2.4	0.5	8.6	3.6	2.0	9.7	9.5	2.2	10	10	10
80	4.5	2.3	0.5	3.5	2.7	1.2	5.2	2.9	0.7	7.2	2.5	1.5	9.5	5.6	1.4	9.8	9.5	3.6	10	10	10
90	9.0	4.5	0.9	7.2	5.5	1.4	9.5	5.5	1.5	9.5	6.1	1.5	9.5	9.5	1.5	10	9.5	7.4	10	10	10
95		9.0	1.8		8.0	1.5		9.5	2.1		9.5	2.9		9.5	7.0		9.5	9.5		10	10
99			9.0			1.8			8.5			5.5			9.4			9.5			10

*Sample:Function ratio (A) 1:9, (B)1:19, (C)1:99.

### Polystyrene (PS) rigid particle separation

**Figure 2:**
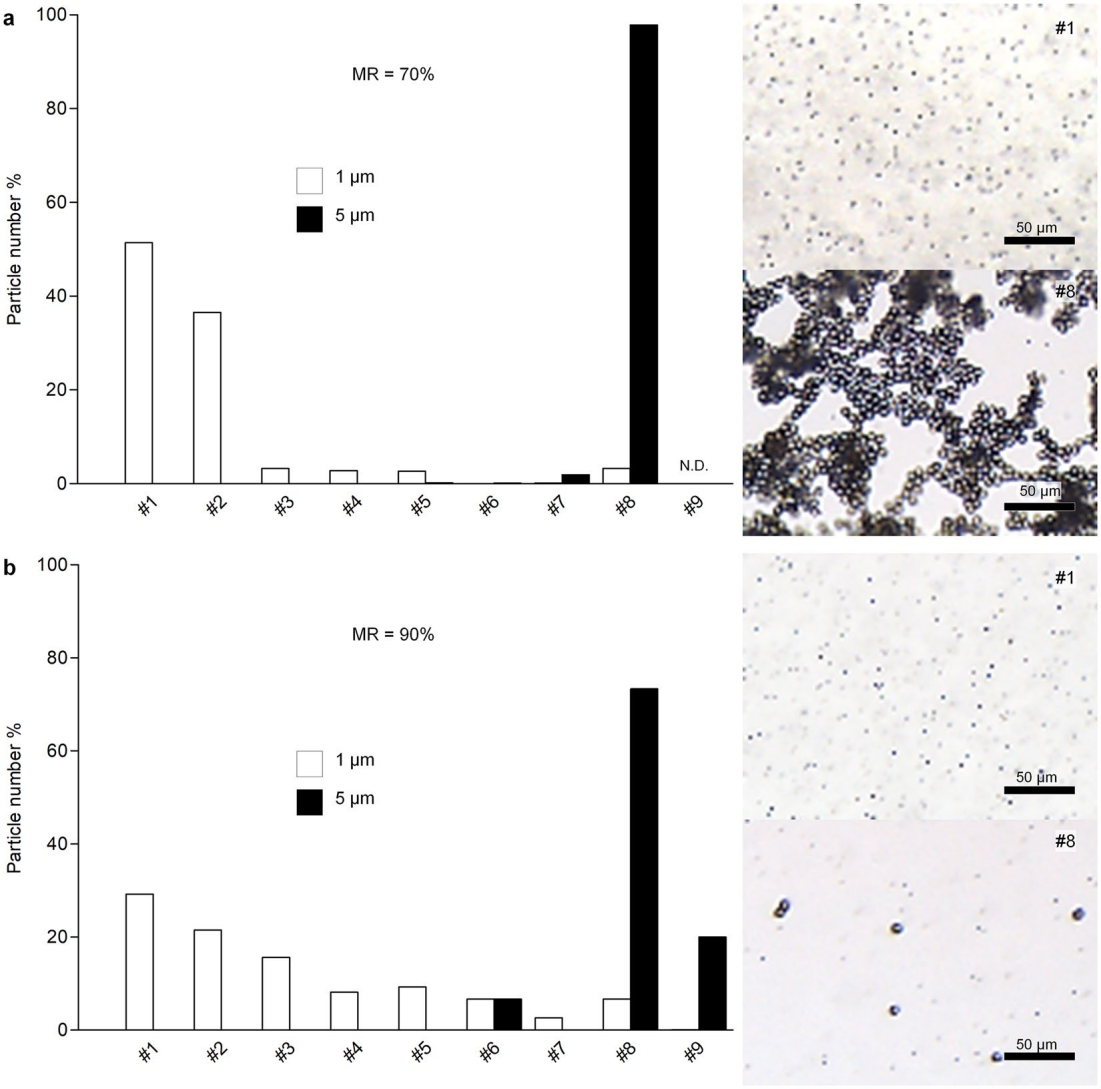
Effect of magnification on particle separation. Mixture of 1 and 5 μm PS microparticles were separated under different magnification ratio (MR), (**a**) 70% (b) 90%. Most of the 5 μm particles are collected at #8 outlet at (**a**) and clearly sorted from 1 μm particles. (**b**) On increasing MR up to 90%, 1 μm particles shift to the high-numbered outlets. More magnification not only spread smaller particles toward more outlets but also moved most of bigger particles moved over the #8 outlet. N.D. represents ‘not detected’.

PS microparticles of various sizes was chosen as model particles to confirm the separation ability of the designed microchannel. Figure 2 and Supplementary Fig. S3 explains how the MR regulates separation of micro-sized PS particles by changing outlet positions of particles. Applying magnification flow induced particles’ shifting to the high-numbered outlets (Supplementary Fig. S3). Under the 70% MR, 1 μm particles were detected mostly at outlets #1 and #2, whereas 90% MR migrated the same size particles up to outlet #8 (Fig. 2). Upon reaching a 90% MR, amount of the 5 μm particles was dramatically reduced (Supplementary Fig. S4), which may infer the loss of particles through the magnification channel. Consequently, the MR regulates size-dependent particle separation by changing the outlet positions of particles.

### Size-dependent separation of cell-derived EVs

**Figure 3:**
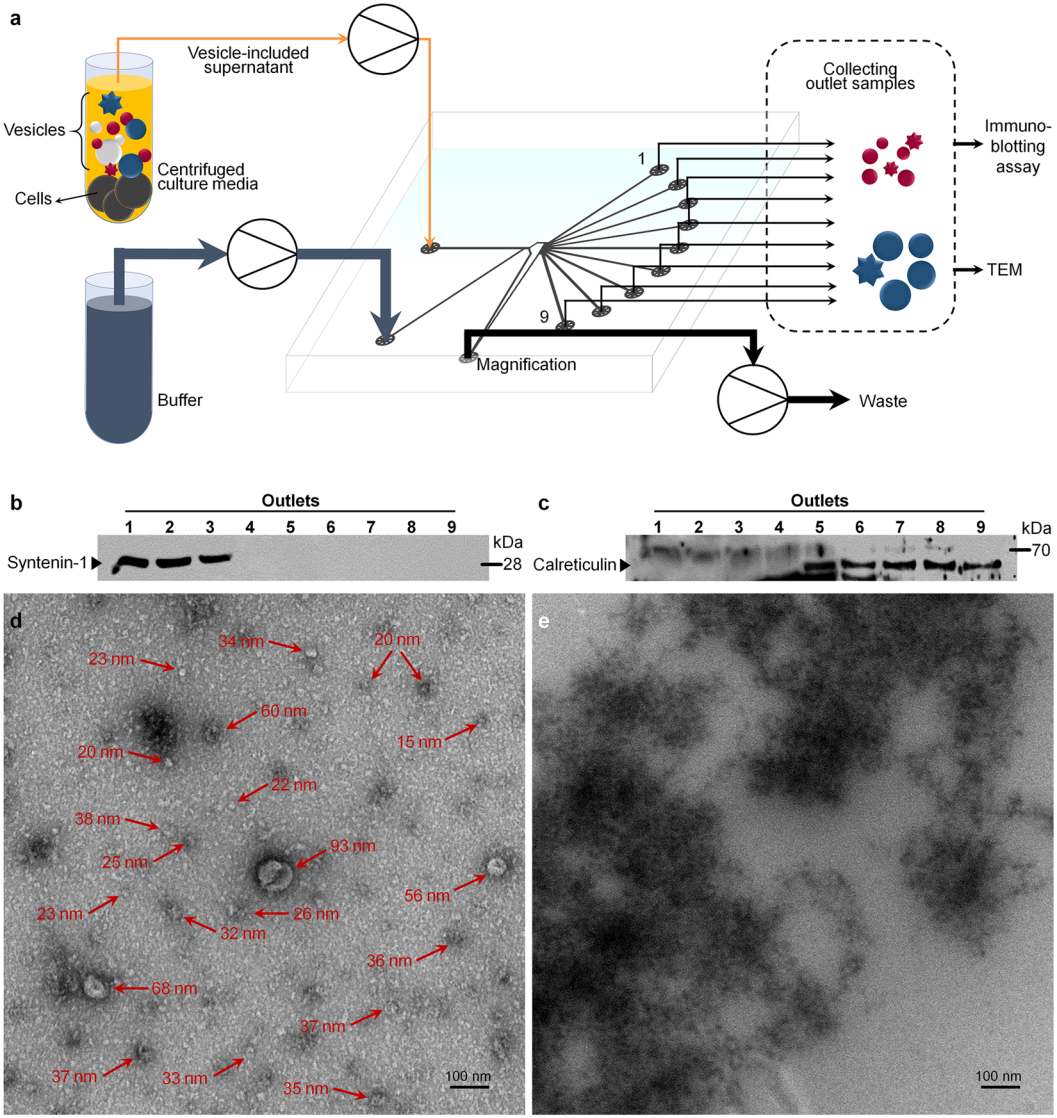
Separation of nanometer- and/or micrometer- biological vesicles from cell culture. (**a**) Separation procedures. After mild centrifugation, cells are discarded and only vesicle-suspended media is concentrated. The enriched vesicles and buffer are separately introduced to Sample and Function channels, respectively. Samples from each outlet are collected and analyzed by immunoblotting assay and TEM. (**b**) (**c**) Immunoblotting results from the separated vesicles with 75% MR are shown as cropped images from full-length western blots. Syntenin-1 and calreticulin antibodies were used as markers for 50–200 nm ranged vesicles, exosomes, and 1–5 μm of apoptotic bodies, respectively. Exosomes are clearly separated through channel #1 to #3. On the other hand, larger microvesicles including apoptotic bodies are collected outlets #5 to #9. (**d**) (**e**) Transmission electron microscopy (TEM) images of vesicles collected from different outlets: (**d**) exosomes at outlet #2 show a distinct cup-shape, which is a specific characteristic of exosomes. Additional vesicles sized down to 20 nm are also detected. Enlarged picture of (**d**) is shown in Supplementary Fig. S7. (**e**) Apoptotic body included aggregates from outlet #8. Round-shape vesicles (arrows at (**d**)) are present in outlet #2 fraction but not shown in outlet #8 fraction, indicating nanometer-sized biological particles were successfully separated from the micron particles including apoptotic bodies.

The specific operating conditions, Sample:Function = 1:9, *Q*_*total*_ = 200 μL/min, MR = 75%, were applied on fabricated microchannel to separate biological vesicles. To prepare the EV mixture, SW620 cells were treated with low dose doxorubicin for 36 h (Supplementary Fig. S6). A low-speed centrifugation step removed cells, after which the supernatants were used as EVs-containing sample (Fig. 3a). Concentrated supernatant and buffer solution were introduced in Sample and Function channel, respectively. After separation proceeded, the suspensions collected from the nine outlets were analyzed by immunoblotting. Syntenin-1 and calreticulin were used as markers for exosomes and apoptotic bodies, respectively. Exosomes were detected at outlets #1 to #3, whereas apoptotic bodies were observed at outlets #5 to #9 (Fig. 3b and c). Additionally, the size of separated EVs was examined by transmission electron microscopy (TEM) analysis. Two representative outlets’ samples (outlet 2: exosome, and outlet 8: apoptotic body) were analyzed by TEM, represented in Fig. 3. Outlet 2 samples showed exosomal cup-shaped morphology and size (30–100 nm), whereas outlet 8 samples showed aggregates including apoptotic bodies and larger sizes (500–2000 nm) (Fig. 3d and e). We confirmed down to 20 nm of biological particles under the electron microscope images of outlet #2 whereas no individual nanoparticles were detected at outlet #8. From the results, EVs appeared to be separated according to their size differences in our EV separation device.

## Discussion

We designed a microfluidic chip for micro- and nano-particle separation including biological vesicles. The microchannel is composed of Sample and Function inlets, nine outlets, and a magnification channel which adjusts particle allocation by altering MR. Similar adaptation of magnification channel has been introduced^25, 26^ for vesicles/particles to move further away from each other. In our case, magnification increased the separation efficiency of vesicles/particles by draining Function flow through the magnification channel. This also minimizes sample dilution, a common feature of microfluidic separation system. We speculate that it should be possible to identify the outlets where particles of a specific size could be collected by controlling the MR. Unwanted or aggregated samples, whose size is larger than others, would be eliminated by flow withdrawal through the magnification channel.

Based on the purpose of the microfluidic separation explained above, determining optimal operational conditions was necessary. Three operation factors, Sample:Function, *Q*_*total*_, and MR, were varied and the status of Sample fluid passing through the outlet channels was observed (Fig. 1e,f, Table 1, and Supplementary Fig. S2). Interestingly, high speed flow, *Q*_*total*_ > 200 μL/min, resulted in spreading of red dye inside all channels regardless of Sample:Function ratio (Supplementary Fig. S2), resulting in larger numbers of colored outlets than the theoretically expected values (Table 1). The turbulence may be generated from the Sample-Function joining region causing dilution of Sample fluid and a considerable amount of sample being lost to the magnification channel. Thus, we determined the maximum flow rate to be 200 μL/min.

The sorting behavior of PS microparticles on the present microchannel was similar to conventional pinched-flow fractionation, but was not confirmed for nanoparticles. In previous reports, synthetic particles smaller than 1 μm had been hard to separate^27^ due to resolution degrading by Brownian motion. However, the flow rate in our system was relatively higher than previously reported ones, allowing us to neglect the diffusion effect. One-dimensional diffusion distance when passing through the channels was calculated as follows: <*d* ^2^> = *kT* × *t*/3*πηR*, where <*d* ^2^>, *k*, *T*, *η*, *R*, and *t* represent mean-square displacement, Boltzmann constant, temperature, dynamic viscosity, particle radius, and diffusion time, respectively. Calculated results were <0.20 and <1.3 μm, less than 0.2% of the channel widths of pinched and broadened regions, respectively. Instead, our system had a relatively large Reynolds number, which was calculated using the equation: Re = (*Inertial force*)/(*Viscous force*) = *ρD*_*H*_ *v*/*η* = 2*ρQ*/*η*(*h* + *w*), where *ρ*, *D*_*H*_, *v*, *η*, *Q*, *h, w* represent fluid density, hydraulic diameter of the microchannel (for a rectangular channel, *D*_*H*_ = 2*hw*/(*h* + *w*)), flow velocity, dynamic viscosity, volumetric flow rate, channel height, and channel width, respectively. The calculated Reynolds numbers for the pinched and broadened region were 27–54 and 1.8–3.5, respectively. This finding implied that flow inside the microchannel was distinguished from the conventional Stokes flow (Re ≪ 1) usually observed in microfluidics. Our microfluidic system managed to maintain a laminar flow with Reynolds numbers less than 2100; however, inertial force could not be ignored for microparticles^28^. Inertia may affect nanoparticle separation. Nevertheless, maintaining a high flow rate (*Q*_*total*_ > 100 μL/min) improves particle separation speed, indicating the ability of the microfluidic device to treat large sample volume.

EVs, especially exosomes, have been developed as markers in diagnostics and therapeutics. Unfortunately, although EVs are good candidates for pharmaceutical development, isolation methods are limited and have limitations. For example, immuno-precipitation methods isolate only specific marker-expressing EVs. Ultracentrifugation methods have a low degree of resolution for size-dependent separation and induce damage in the EVs. Compared with conventional EV isolation methods, our microfluidic-based EV separation system offers fine-tuned EV separation in a size-dependent manner since apoptotic bodies (1–5 μm) were much larger than exosomes (hydrodynamic diameter, 50–200 nm)^4, 29^, and is a flexible platform for targeting different sized EVs. As shown in Supplementary Fig. S5, the size-separation tendency of vesicles was similar to that in the PS particle separation. Even though the particle properties would be different, we confirmed that the vesicle/particle mixtures, whose sizes were approximately 1 μm, could be separated using our system. Moreover, MR is adjusted depending on what kind of target is chosen. We applied distinct dissimilar operational conditions; however, both biological vesicle and PS particles having different size ranges were successfully separated. Since this platform suggests that differently characterized EVs can be isolated, new diagnostic markers and therapeutic candidates could be identified using this platform.

Based on the results above, the suggested separation method covers wide range of biological nanoparticle. Microparticles should be separated under MR < 70% to protect loss of samples through the magnification channel (Fig. 2). Under the same condition of PS particle separation, the number of colored outlets was 2.2. In case of EV separation from the cultured media (Fig. 3), most of biological vesicles have sub-micron sized diameter, which is much smaller than tested PS particles, thus we adapted the condition whose number of colored outlets was approximately 9.0. From this compared results, we propose that the operational conditions of higher number of colored outlets should be set for smaller vesicle/particle separation. Consequently, vesicle/particle of various sizes, from sub-micron to tens of micron in diameter, could be sorted in single microchannel by controlling MR only. In common, sample pretreatment has been accompanied in order to remove aggregated impurities that block the channel and fail the chip operation. Our suggesting microchannel can eliminate pretreatment step by discarding larger impurities such as cells and aggregated vesicles through magnification channel as well as separate specific size particles through nine outlets simultaneously.

In summary, noninvasive microfluidic particle separation was performed to separate micro- and nano-particles by size, and eventually separate extracellular vesicles. This microchip contains a magnification channel; hence, particle allocation inside the channel and/or toward the outlets is controllable according to the characteristics of sample fluid and contained particles through MR change. We successfully separated nanometer-sized exosomes and apoptotic bodies at outlets #1 to #3 and outlets #5 to #9, respectively. Biological differences of the vesicles were confirmed by immunoblotting assay and TEM image.

The present system and methodology provided new ways of biological nanovesicle separation in microfluidic formats especially Reynolds number ranging in 1 to 100 minimizing diffusion effects. Efforts to understand specific positioning of vesicles or particles in the micro channels should be continued. In the meantime, separation of particles under increased operation speeds could enhance the separation efficiencies. Indeed, we expect that the separation of nano- to micro-sized particles and vesicles from biological samples including blood without any pretreatments based on the present results have great potentials to contribute in the advancement of biological pharmaceutical and diagnostic sciences.

## Methods

### Microchannel design and microchip fabrication

The PDMS-based microchannel was prepared following previously reported methods^14, 30^. The microchannel was designed using AutoCAD (Autodesk Inc., San Rafael, CA) and printed as a 25,000 dpi photomask (Microtech, Korea). After spin-coating and baking the SU-8 photoresist (Microchem, Westborough, MA) on a 4 in silicon wafer, the photomask was placed, UV-exposed, and developed. Next, PDMS (Sylgard 184®, Dow Corning, MI) was mixed with the elastomer at a ratio of 10:1, poured over the SU-8 microchannel mold, and cured for 40 min at 80 °C in the oven. All inlets and outlets on the PDMS chip were punched using a micro-puncher (Syneo, Angleton, TX). PDMS, whose mixing ratio with the elastomer was 20:1, was spin-coated on a clean glass slide with a thickness of 10–20 μm, followed by curing for 20 min at 80 °C. The punched PDMS chip was then placed on the partially cured PDMS coated glass slide and curing was applied for a further 18 h.

### Preparation of PS particle suspension and biosamples

Polystyrene (PS) microparticles and nanoparticles were purchased from Sigma (St. Louis, MO). The mixing concentrations of the PS particles were 0.1% (w/w) for 1 μm and 0.9% (w/w) for 5 μm particles. All solutions were suspended in distilled water and mixed immediately prior to beginning the experiment. The apoptotic bodies were prepared by incubating SW620 (ATCC, Manassas, VA) cells with 2 μM doxorubicin (Sigma-Aldrich, St. Louis, MO) in RPMI-1640 medium containing 1% penicillin-streptomycin (Hyclone, Logan, UT) for 36 h. The culture medium was harvested and cells were removed by centrifugation at 400 *g* for 10 min. The supernatant was centrifuged at 2,000 *g* for 20 min and the pellet was resuspended in PBS for the experiment. Exosome was also obtained from SW620 cell culture media without doxorubicin. After cells and cell debris removal by centrifugation, the supernatant was centrifuged additionally at 10,000 *g* for 20 min. The supernatant were concentrated with 50 K Amicon centrifugal filters (Millipore, Billerica, MA) for harvest exosomes. Details are explained in Supplementary Fig. S6.

### Experimental operating conditions

Three syringe pumps (Fusion 100 and 200, Chemyx, Stafford, TX) were used to generate the microflow; one for infusion of vesicle/particle suspended sample, one for infusion of Dulbecco’s phosphate buffered saline (DPBS, Welgene, Republic of Korea)- based Function flow, one for withdrawal flow from the magnification channel. *Q*_*total*_ was adjusted to 200 μL/min (for biological sample separation, MR = 75%) and 100 μL/min (for PS particle separation, various MR applied). The input ratios of Sample:Function of biological samples’ and PS particles’ separation were 20:180 and 5:95, respectively. Tygon Microbore tubings (Cole-Parmer, Vernon Hills, IL) connected syringes to micropins (New England Small Tube, Litchfield, NH), which were fit to the punched holes. The ends of the connected tubing were dipped in distilled water and the water level was adjusted to that at the surface, to allow for sampling from the outlets and render negligible the effect of pressure differences at the outlets. Distilled water was applied to flush air bubbles from inside the channel, following which particle separation could proceed.

### Calculation of the theoretical value of Number of colored outlets

To compare the flow pattern of Sample fluid (red dye) on Fig. 1e and Supplementary Fig. S2, theoretically expected values of Number of colored outlets were calculated. Assuming no mixing between the Sample and Function fluid, it is expected that only Function flow is withdrawn through the magnification channel when Function flow rate is similar to or higher than Magnification flow rate (*Q*_*F*_ ≥ *Q*_*M*_). We assumed that the remained fluid is distributed evenly to the 9 outlets, thus (*Q*_*total*_ − *Q*_*M*_)/*N* = *Q*_*S*_/*N*_*colored*_, where *Q*_*total*_, *Q*_*S*_, *N*, and *N*_*colored*_ are total flow rate, Sample flow rate, total outlet numbers (fixed as 9 in the present work), and Number of colored outlets, respectively. MR (Magnification ratio) [%] = 100*Q*_*M*_/*Q*_*total*_, thus, Number of colored outlets can be derived as follow:

$${N}_{colored}=\frac{N{Q}_{S}}{({Q}_{total}-{Q}_{M})}=\frac{100N{Q}_{S}}{{Q}_{total}(100-MR)}$$

Under the 1:19 Sample:Function ratio with *Q*_*total*_ = 100 μL/min and MR = 80%, *Q*_*S*_ = 5 μL/min, thus, *N*_*colored*_ = 2.25. The calculated values for every condition are summarized in Table 1.

### Analysis of outlet samples

The samples were collected in nine tubes and magnification syringe, and were analyzed by several methods (the flowchart for the overall procedure is presented in Supplementary Fig. S1). For micro-sized PS particles, the separated and collected samples were placed in a 96-well plate, and the number of particles was counted using an optical microscope. For biological vesicles, exosomes and apoptotic bodies were distinguished by immunoblotting assay. Proteins and EVs were precipitated from outlet samples using trichloroacetic acid (TCA, Sigma-Aldrich, St. Louis, MO). After neutralization with 100 mM HEPES (pH 8.0), precipitates were loaded on SDS-PAGE and transferred to PVDF membrane (Millipore, Billerica, MA) for immunoblotting. Antibodies against syntenin-1 (Santa Cruz, Dallas, TX) and calreticulin (Thermo Scientific, Lafayette, CO) were used to probe for exosomes and apoptotic bodies, respectively. These vesicles were confirmed by electron microscopy imaging. Isolated nanoparticles were diluted 5 fold in PBS, then 5 µL of the sample solution was immediately (~ 5 sec) applied to a carbon-coated grid that had been glow-discharged (Harrick Plasma) for 3 min in air, and the grid was negatively stained using 1% uranyl acetate. The same procedure was used for all negatively stained specimens. The stained vesicles were imaged under a Hitachi H-7600 (Hitachi, Tokyo, Japan) transmission electron microscope at 80 kV.

### Mean particle size calculation of each sample

As explained in Supplementary Fig. S1, all analyzed data were re-calculated to determine the mean particle size of each outlet sample and to identify the separation profiles (Figs 2 and 3, and Supplementary Fig. S3). For PS microparticle size calculation, the following equation was used: (Mean particle size) = *D*_*a*_ *p*_*a*_ + *D*_*b*_ *p*_*b*_/(*p*_*a*_ + *p*_*b*_), where *D*_*i*_ and *p*_*i*_ are particle diameter and particle number percentage of ‘*i*’ μm particles (*i* = 1, 5, 10), respectively. For example, if the number of 1 μm particle is 10 times higher than 5 μm particles, mean particle size is calculated to be 1.36 μm. For biological vesicles, vesicle types and expected particle size range were confirmed from the immunoblotting data and the size-represented reference^6^, respectively. To compare the separation tendencies shown in Supplementary Fig. S5b, larger size position at y-axis was decided for apoptotic body.

## Electronic supplementary material

Supplementary information
