# Supplementary material for: Separation of extracellular nanovesicles and apoptotic bodies from cancer cell culture broth using tunable microfluidic systems
Source: Sci Rep. 2017 Aug 30;7:9907. doi: 10.1038/s41598-017-08826-w (PMC5577194; doi:10.1038/s41598-017-08826-w)
Supplement: Supplementary file 1 — Supplementary information [file 41598_2017_8826_MOESM1_ESM.pdf]

Supplementary information

Separation of extracellular nanovesicles and apoptotic bodies from cancer cell culture broth using tunable microfluidic systems

Soojeong Shin[1§], Daeyoung Han[2,3§], Min Chul Park[2,4§], Ji Young Mun[5,6], Jonghoon Choi[7], Honggu Chun[8], Sunghoon Kim[2,3]*, and Jong Wook Hong[1,9]*

* To whom correspondence should be addressed.
§ These authors contributed equally to this work.

[1]*Department of Bionano Engineering, Hanyang University, Ansan 15588, Korea*
[2]*Medicinal Bioconvergence Research Center, Seoul National University, Seoul 151-742, Korea*
[3]*Department of Molecular Medicine and Biopharmaceutical Sciences, Graduate School of Convergence Technology, College of Pharmacy, Seoul National University, Seoul 08826, Korea*
[4]*Advanced Institutes of Convergence Technology, Seoul National University, Suwon 443-270, Korea*
[5]*Department of Biomedical Laboratory Science, College of Health Sciences, Eulji University, Seongnam 13135, Korea*
[6]*BK21 Plus Program, Department of Senior Healthcare, Graduate School, Eulji University, Daejeon 34824, Korea*
[7]*School of Integrative Engineering, Chung-Ang University, Seoul 06974, Korea*
[8]*Department of Biomedical Engineering, Korea University, Seoul 08826, Korea*
[9]*Department of Bionano Technology, Graduate School, Hanyang University, Seoul 04763, Korea*

CONTENTS

Supplementary Figure S1. Flow chart for data analysis and enumeration of inserted or collected samples. ······ 2

Supplementary Figure S2. Effect of total flow rate (Q_{total}) and Magnification ratio (MR) on sample flow expanding is evaluated under the different Sample-to-function ratio; (a) 1:9, (b) 1:19, (c) 1:99. ······ 3

Supplementary Figure S3. PS microparticle sorting tendencies under the different MRs, (a) 0% and (b) 70%, for 1 and 10 μm particle separation. Magnification flow gives overall shift of microparticles toward the higher-number outlets. ······ 4

Supplementary Figure S4. Optical microscopic pictures of samples collected in each outlet after PS microparticle separation. ······ 5

Supplementary Figure S5. Comparison of size separation tendencies under appropriate MRs in the microfluidic separation chip. ······ 6

Supplementary Figure S6. Scheme for extracellular vesicle preparation. ······ 7

Supplementary Figure S7. Enlarged picture of **Fig. 3(d)**. ······ 8

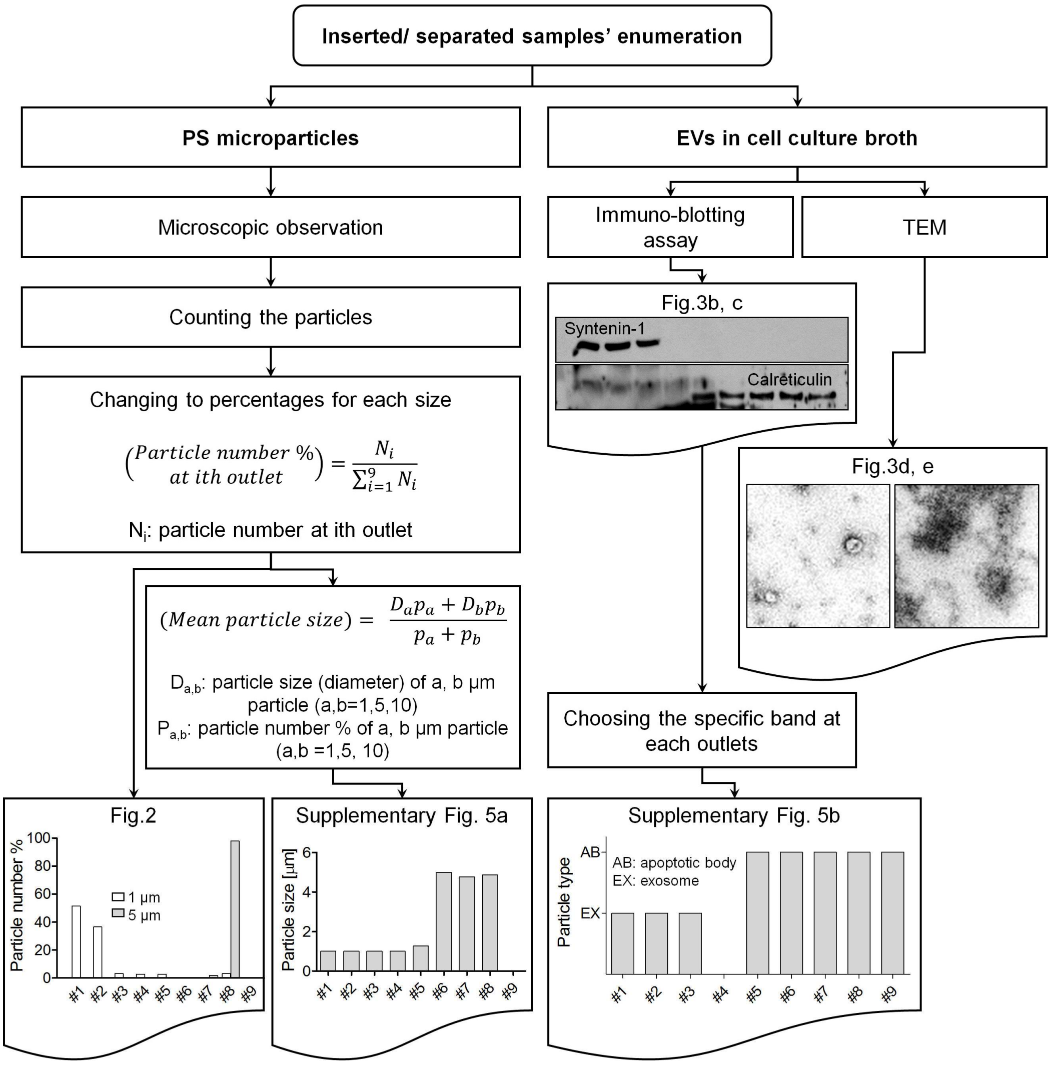

Supplementary Figure S1. Flow chart for data analysis and enumeration of inserted or collected samples.

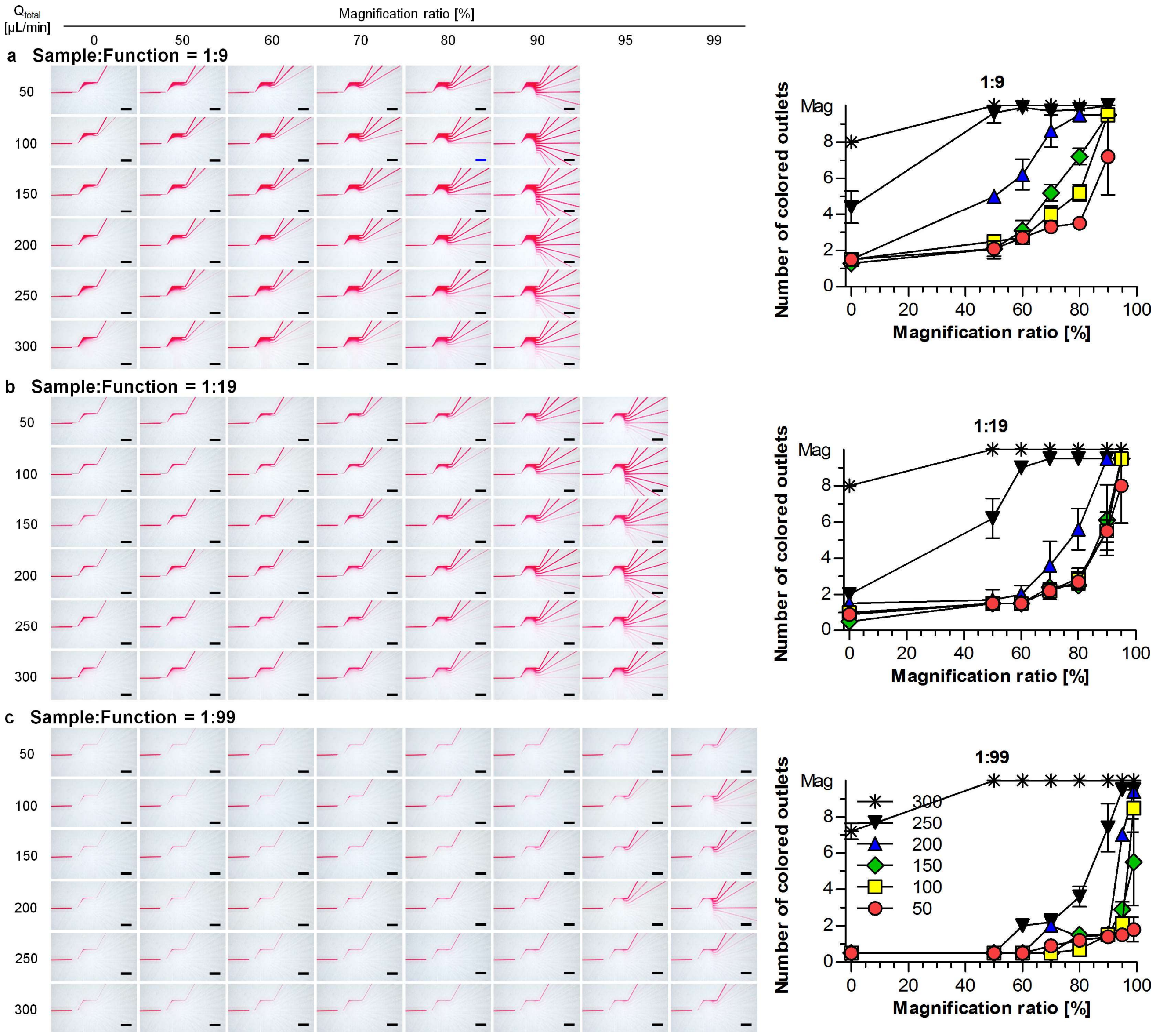

Supplementary Figure S2. Effect of total flow rate (Q_{total}) and magnification ratio (MR) on sample flow expanding is evaluated under the different sample-to-function ratio; (a) 1:9, (b) 1:19, (c) 1:99. Scale bar = 1 cm. Red dye represents sample flow passing through the channel. We count the number of reddish outlet channels and correlated with MR, shown as a graph at the right of the figure. Portion of function fluid increment indicates that less sample is injected; thus, the reddish portion in channel is the highest at (a) and lowest at (c). Stronger withdrawal at a higher MR induces sample flow expansion up to #9 outlet; however, too much withdrawal, similar or higher magnification flow rate than function flow rate, causes loss of sample through magnification channel. Less withdrawal, meaning much lower magnification flow rate than function flow rate, shows lack of expansion of sample flow. Q_{total} > 200 µL/min induces voltex effect; finally the red dye spreads to all channels. The summarized data are displayed as 3-dimensional landscaping graphs in **Fig. 1f**.

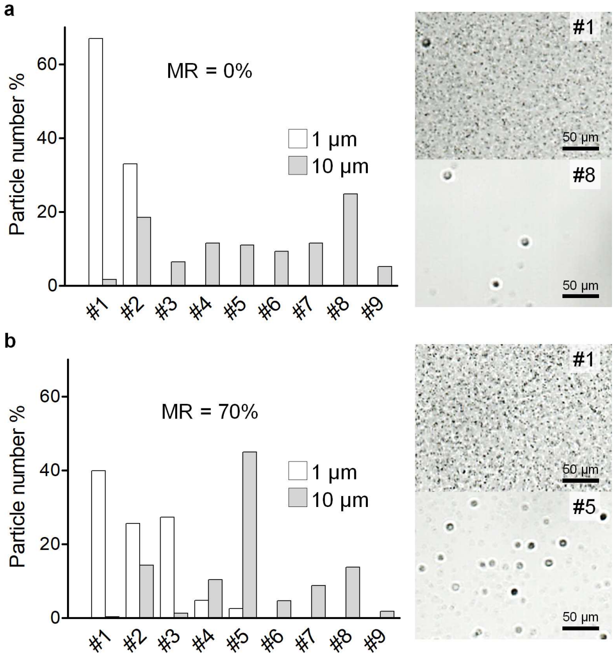

Supplementary Figure S3. PS microparticle sorting tendencies under the different MRs, (a) 0% and (b) 70%, for 1 and 10 μm particle separation. Magnification flow gives overall shift of microparticles toward the higher-number outlets.

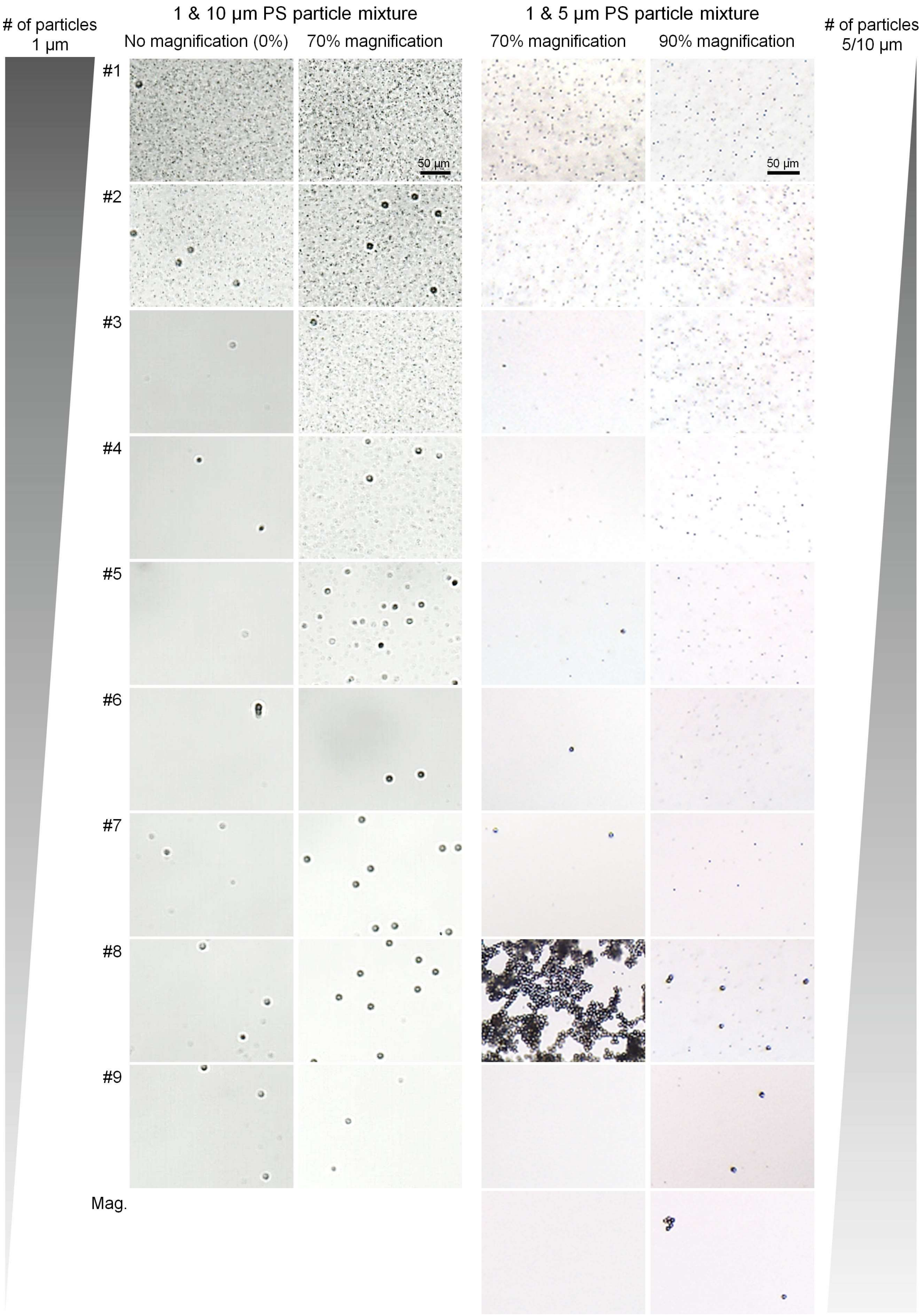

Supplementary Figure S4. Optical microscopic pictures of samples collected in each outlet after PS microparticle separation. These pictures were enumerated quantitatively to the graphs in **Figure 2** and **Supplementary Figure S3**.

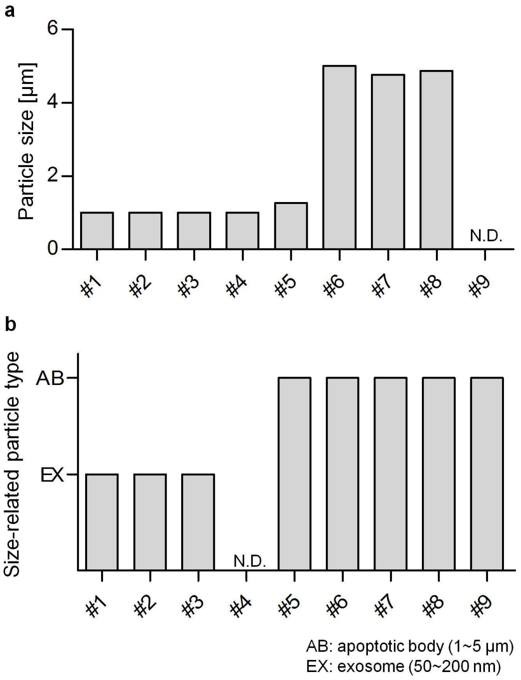

Supplementary Figure S5. Comparison of size separation tendencies under different MRs of 70 and 75% in the microfluidic chip. Both rigid PS particles and soft biological vesicles are sorted out by size. The graphs are rearranged from (a) **Fig. 2a**; (b) **Fig. 3b and c**. Detailed replotting procedure is explained in **Supplementary Fig. S1**. N.D. represents 'not detected'.

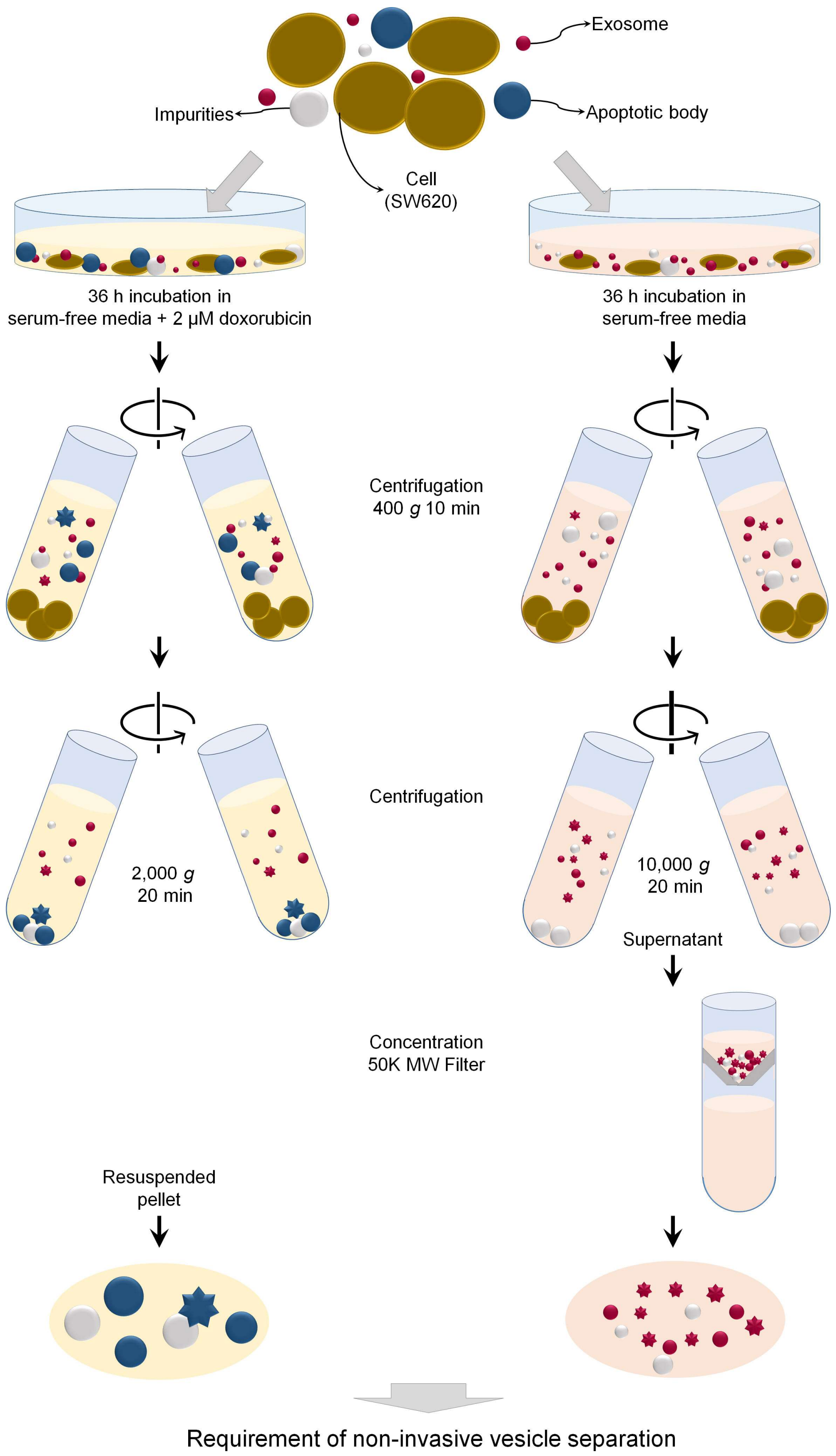

Supplementary Figure S6. Scheme for extracellular vesicle preparation (not to scale).

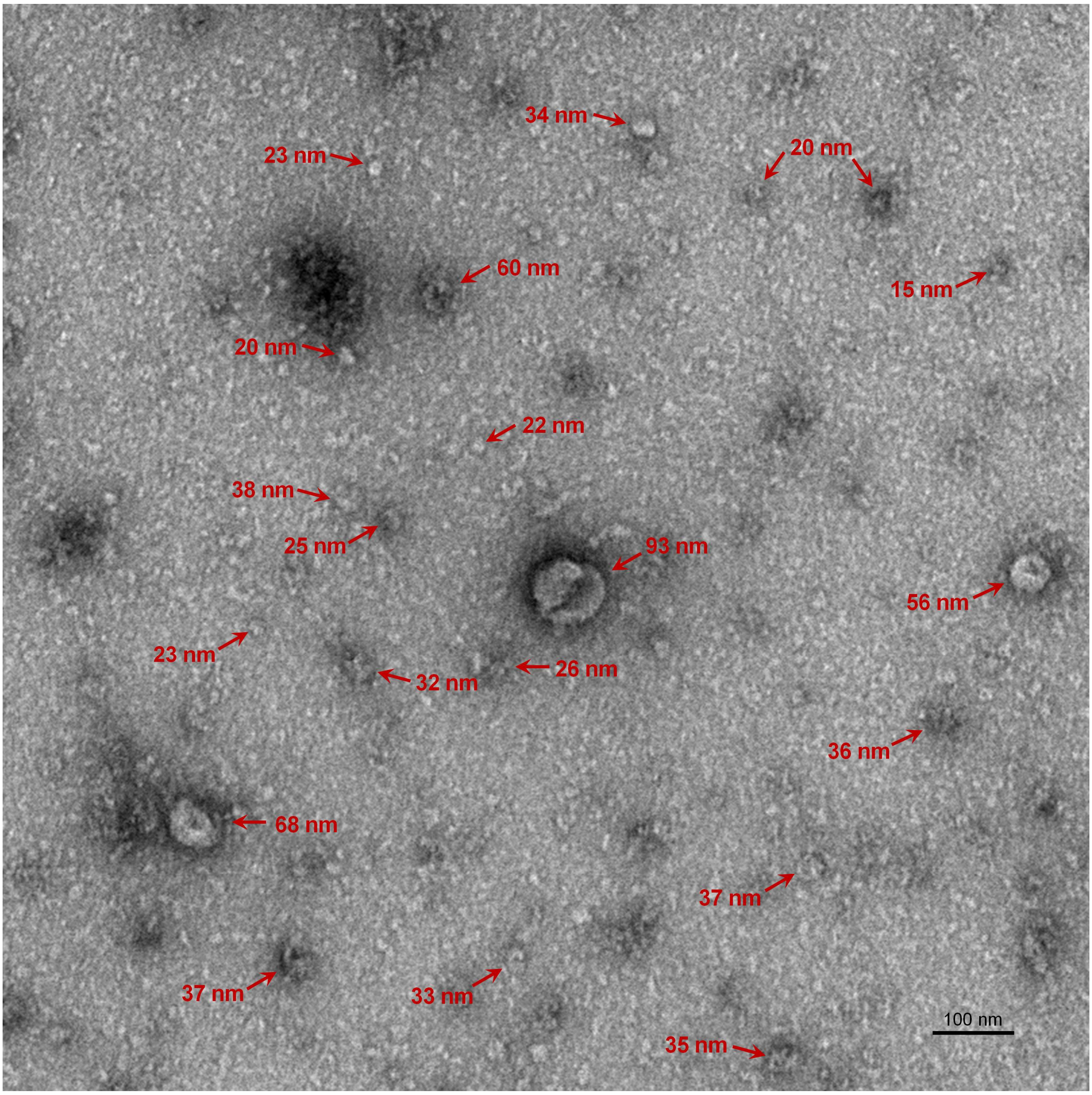

Supplementary Figure S7. Enlarged picture of **Fig. 3(d)**.
